# A Grammar Inference Approach for Predicting Kinase Specific Phosphorylation Sites

**DOI:** 10.1371/journal.pone.0122294

**Published:** 2015-04-17

**Authors:** Sutapa Datta, Subhasis Mukhopadhyay

**Affiliations:** Department of Biophysics, Molecular Biology and Bioinformatics and Distributed Information Centre for Bioinformatics, University of Calcutta, Kolkata, West Bengal, India; Huazhong University of Science and Technology, CHINA

## Abstract

Kinase mediated phosphorylation site detection is the key mechanism of post translational mechanism that plays an important role in regulating various cellular processes and phenotypes. Many diseases, like cancer are related with the signaling defects which are associated with protein phosphorylation. Characterizing the protein kinases and their substrates enhances our ability to understand the mechanism of protein phosphorylation and extends our knowledge of signaling network; thereby helping us to treat such diseases. Experimental methods for predicting phosphorylation sites are labour intensive and expensive. Also, manifold increase of protein sequences in the databanks over the years necessitates the improvement of high speed and accurate computational methods for predicting phosphorylation sites in protein sequences. Till date, a number of computational methods have been proposed by various researchers in predicting phosphorylation sites, but there remains much scope of improvement. In this communication, we present a simple and novel method based on Grammatical Inference (GI) approach to automate the prediction of kinase specific phosphorylation sites. In this regard, we have used a popular GI algorithm Alergia to infer Deterministic Stochastic Finite State Automata (DSFA) which equally represents the regular grammar corresponding to the phosphorylation sites. Extensive experiments on several datasets generated by us reveal that, our inferred grammar successfully predicts phosphorylation sites in a kinase specific manner. It performs significantly better when compared with the other existing phosphorylation site prediction methods. We have also compared our inferred DSFA with two other GI inference algorithms. The DSFA generated by our method performs superior which indicates that our method is robust and has a potential for predicting the phosphorylation sites in a kinase specific manner.

## Introduction

Protein phosphorylation is one of the most important ubiquitous post-translational modifications. Protein phosphorylation occurs due to the addition of covalently bound phosphate group to certain receptor residues i.e., Serine (S), Threonine (T), Tyrosine (Y) and Histidine (H) in the substrate sequence. Protein phosphorylation plays a major role in various critical cellular phenomenon such as metabolism [1], cell signaling [2,3], cellular proliferation [3] and apoptosis [4] and found in almost all the organisms from prokaryotes to eukaryotes. About 30%-50% of all eukaryotic proteins are known to undergo protein phosphorylation [5].

Phosphorylation takes place through a set of enzymes called kinases, which constitutes one of the largest known protein super families. About 1.7% of all the human genes encode as many as 518 different types of kinases and they are classified into a hierarchical manner with 10 groups, 134 families and 201 subfamilies primarily based on the homology of their catalytic domains [6]. Therefore, the identification of phosphorylation sites, especially in a kinase-specific manner, is necessary for understanding the molecular mechanisms of phosphorylation as well as elucidating the dynamic interactions between protein kinases (PKs) and their substrates. Although mass spectrometric techniques has been widely used in detecting the phosphorylation sites in a high-throughput manner, but this method is rather cost and labor intensive. Also, ever increasing number of protein sequences in the data banks necessitates the development of computational methods for reliably predicting phosphorylation sites in the protein sequences as fast as possible. Many *in silico* methods have been proposed to predict kinase specific phosphorylation sites, such as Scansite [7], KinasePhos [8], NetPhosK [9], PPSP [10], GPS [11,12], Postmod [13], BAE [14], AMS 4.0Server [15], Metapred [16] and a method that we have developed earlier [17]. These methods predict the phosphorylation sites in a kinase specific manner. The details of these methods are discussed in the review paper by Trost *et al*. [18]. Most of these methods are based on machine learning techniques using a single classifier. In the recent years, researchers are concentrating upon using ensemble mechanism instead of a single classifier for predicting protein phosphorylation sites [14–15, 17]. Another method Musite is considered for large scale predictions of both non-kinase and kinase-specific phosphorylation sites [19]. RegPhos and KinomeXplorer are two latest tools, aimed to explore kinase signaling networks [20, 21]. Recently in 2014, Suo *et al*. have proposed a method PSEA for predicting kinase specific phosphorylation sites as well as for analyzing the types of kinases corresponding to all disease-related phosphorylation substrates [22]. But all of these methods require a good data encoding scheme as it plays a crucial role in affecting the performance of the classifiers. Moreover, a specific type of feature encoding scheme for precisely predicting phosphorylation sites of a protein sequence is not fully exploited. Therefore, no single feature encoding scheme can be expected to absolutely differentiate the phosphorylation from non-phosphorylation sites for all the kinases. Also, these methods require an *a priori* knowledge about the computational models of phosphorylation sites to permit automatic annotation.

**Fig 1:**
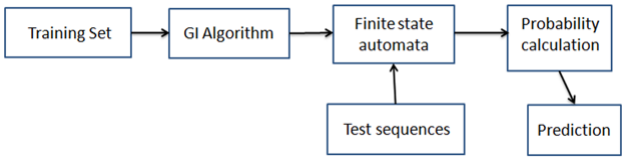
Block diagram of a general Grammar Inference methodology.

In our study, we have proposed a new method to support the *de novo* discovery of kinase specific phosphorylation sites based upon computational grammar. Various methods based on Computational grammars have been proposed so far for modelling and predicting various types of biological sequences such as promoter region of human [23], transcription binding site [24], associating genes with their regulatory sequences [25], predicting RNA folding [26], secondary structure of RNA molecule [27–29], genes and biological sequences [30,31], syntactic model to design genetic constructs [32] and new antimicrobial peptides [33]. Nowadays, Grammar Inference (GI) is becoming an active field of research in the area of computational grammar [34]. GI is a specific type of inductive inference, which takes into account a set of sample data to obtain a model consistent with the available data. The resulting model obtained through GI method using a set of sample strings represents a formal grammar which contains all common features of the strings. GI method is being used for predicting various biological sequences, such as larger than gene structure [35], functional motifs, such as coiled coil domains in protein sequences [36], transmembrane domains [37], various protein sequences [38], etc. The choice of GI as a method confers us with two distinct advantages over other methods having a different flavor: **(a)** GI doesn’t require any pre-existing biological knowledge and **(b)** no data encoding is required for a GI. A general framework of GI methodology is shown in Fig 1. In our study, we have used GI method to infer the regular grammar corresponding to the phosphorylation sites of protein sequences. From previous studies we came to know that substrates of a specific kinase show a particular sequences pattern around the phosphorylation sites [39, 40]. For example, PKA kinase has a preference to identify the substrate sites with basic amino acids (Arginine, Lysine or Histidine) at -2 or -3 positions relative to the phosphorylation sites considered as position 0 [40]. Many methods have been developed to infer the sequence motifs near the phosphorylation sites [7, 39, 40, 41]. Our method is based on the idea of identifying recurring patterns hidden in the phosphorylation sites from a set of training samples and representing them as grammatical models. The generated grammar is then used for predicting unknown phosphorylation sites. A grammar can equally be represented by means of an automaton. Instead of inferring grammar rules, in our present study, we have inferred the Deterministic Stochastic Finite State Automata (DSFA) using Alergia algorithm for predicting the kinase specific phosphorylation sites. The method we have presented here is an unsupervised learning method based on Prefix Tree Automaton (PTA). At the very outset, our method first constructs a PTA based on the training samples. In the next step, the similar states of the PTA are merged in an iterative process to obtain a stochastic finite state automaton. The resulting automata will allow processing of unknown protein sequences for deciding upon whether to accept or to reject the sequences. The point to be noted here is that it can accept a wider range of input sequences than what may be present in the training set.

## Materials and Methods

### Materials

In order to evaluate the performance of our proposed method and to compare it with other existing methods, we have extracted the phosphorylation sites from Phospho.ELM database (version 9.0) [42]. Experimentally validated phosphorylation sites of eukaryotic cells for 299 types of different kinases are curated in Phospho.ELM database. Version 9.0 of this database contains 8718 proteins from different vertebrate species covering 31,754 serine, 7,449 threonine and 3,370 tyrosine instances. Each entry in the database provides information about the substrate proteins along with the exact positions of the residues phosphorylated by a given kinase. In our study, we have considered the kinase families having at least 100 known and experimentally validated phosphorylation sites. We have chosen four serine/threonine (S/T) kinase families, PKA, PKC, CK2 and MAPK for vertebrates only. 21mer sequences including 10bp upstream to 10bp downstream from the phosphorylation site with the phosphorylated residue at the central position i.e., at the position 11 are extracted. In case the Phosphorylated residue (S/T) which appears in the first or in the last 10 residue, that is, if the distances between the phosphorylated residue and N or C terminal is less than 10-mer, an extra residue X (where X denotes any residue) is added before the first residue or after the last residue of the sequence in order to keep the protein sequence size at 21mer and phosphorylated residue at the center i.e., at the position 11.

Negative dataset is prepared by taking the 21mer protein sequences centring all the non-phosphorylated S, T residues of the substrate proteins corresponding to the nine kinase families mentioned above. We have taken non-phosphorylated S/T residues as negative control for the serine/threonine kinases that is, for PKA, PKC, CK2 and MAPK kinase families. In order to avoid the overestimation during experimentation, highly homologous sequences (i.e., sequences having more than 60% identity) are discarded from positive and negative datasets by using CD-HIT clustering program [43].

Moreover, in the context of the multiple kinase families, the number of known positive phosphorylation sites is much less than the negative sites i.e., non-phosphorylation sites, resulting in an imbalanced dataset. As there are far more non-phosphorylated S/T residues than phosphorylated ones, it is not viable to take up the whole non-phosphorylated sites as negative instance. Furthermore, in the real world, there are much more negative cases than positive cases. Hence it would be more practical to take a significant disparity in positive to negative ratio for evaluating the performance of our method in a more credible manner. So in our present study, the ratio of positive instances to negative instances is kept at 1:10 to avoid any biased prediction.

### Method

The main goal of our method is to construct the regular grammar corresponding to the phosphorylation sites. The problem of learning of a regular grammar can be reduced to that of the inference of finite state automata. The inference of finite state automata is widely studied by the GI community. Various GI methods have been proposed so far for the successful induction of finite state automata. In our work, we have inferred the deterministic finite state automata (DFA) that can recognize the phosphorylation sites of the protein sequences in a kinase specific manner. To achieve this goal, a well known GI algorithm has been used to infer automata from a set of training samples. In the test phase, when an unknown sequence is given as input, the generated automata decide whether the sequence contains a phosphorylation site or not.

### Notation and definition

Let Σ be the set of alphabet and Σ* be the set of words generated over the set of alphabet Σ. Then a language L can be any subset of Σ* i.e., the subset of the set of words and can be written as L = {x | xϵΣ* where x is any word}

If G is the grammar corresponding to the language L, grammar is denoted as G = (N, Σ, P, S) where N is the set of nonterminal symbols, Σ is the set of terminal symbols or the set of alphabets, P is the set of production rules and S is the start symbol. The language L can be generated successively by rewriting the production rules starting from the start symbol S.

A regular grammar is a type of formal grammar that describes and generates a regular language. Regular grammar is a 4-tuple (N, Σ, P, S) such that the production rules in P are of the following forms:

1. *B* → *a*, where *B* ϵ *N* and *a* ϵ Σ
2. *B* → *aA* | *Aa*, where *B*, *A* ϵ*N* and *a* ϵ Σ
3. *B* → *ε*, where *B* ϵ *N* and *ε* denotes the empty string i.e., string of length 0.

Regular grammar can equally be represented by Deterministic finite state automata (DFA). Most of the previous work in regular grammar inference has chosen DFA to represent the target regular grammar. A DFA is a virtual machine, formally represented by 5-tuple (Q, Σ, δ, q_0_, F) where Q is the set of states, Σ is the set of symbols or alphabets, q_0_ ϵ Q is the start state, F ϵQ is the set of final accepting state, δ is the transition function *Q* × Σ→*Q δ*(*q*,*a*) denotes the state reached from the state q on reading the input symbol *a* of Q. The automata process an input string and decide whether to or not to accept the string. For reading an input string, DFA must reach to any final accepting state beginning from the start state. Therefore, a successful path in an automata is a sequence of transitions (*q*_0_,*a*_1_,*q*_1_),(*q*_0_,*a*_2_,*q*_2_)…(*q*_*n*-1_,*x*_*n*_,*q*_*n*_), where *q*_*n* ∈_ *F*, *a*_*i*_ ϵ Σ* and 1 ≤ *i* ≤ *n*: *q*_*i*_ ϵ *Q*

### Grammar inference

We have addressed the prediction of kinase specific phosphorylation sites through a DFA. For this, we have tried to construct DFA from a set of amino acid sequences that contain the phosphorylation sites. Now, given an unknown sequence, the constructed DFA would only accept the sequences having phosphorylation sites. To infer a DFA, initially a Prefix Tree Automaton (PTA) is constructed from a set of positive samples (S^+^) using the PTA algorithm.

**Fig 2:**
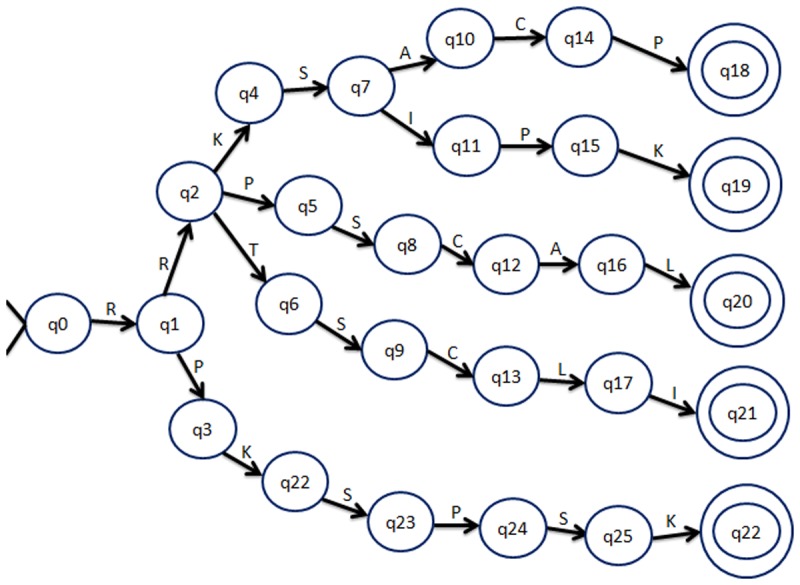
A Prefix Tree Automaton (PTA) generated over Five protein sequences: i) RRKSACP, ii) RRKSIPK, iii) RRPSCAL, iv) RRTSCLI, v) RPKSPSK.

**Fig 3:**
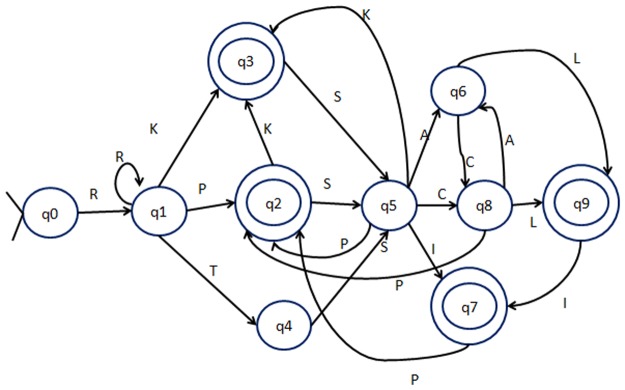
The Deterministic Finite State Automaton (DFA) constructed from the PTA by state merging method.

PTA algorithm is an unsupervised relational learner that infers grammars from a set of unlabelled samples. PTA algorithm works by constructing a PTA, which is also a DFA with separate paths from the start state to the final accepting state for each string in S^+^. Each of the DFA represents an input token (in our case, each amino acid). PTA accepts the strings from S^+^ only. In the next step, similar states of the PTA are merged in an iterative way until a minimum similarity threshold is reached. In each iteration, the similarity between every two states is calculated and the most similar pair of states is merged to obtain the final minimized DFA. Fig 2 shows the diagram of a PTA and Fig 3 represents the final DFA generated over five sample sequences.

In this study, instead of inferring simple regular grammar, we have inferred stochastic regular grammar. Towards this endeavor, we have inducted the corresponding Deterministic stochastic finite state automata (DSFA). A DSFA is a 4-tuple and is defined as *A = (Q*, *Σ*, *q*_*1*_, *π)*, where Q is the finite set of N states, Σ is the finite set of symbols or alphabets, *q*_*1*_ is the initial state and π is the set of probability matrices. *P*_*ij*_ *(a)* is the probability of transition from state *i* to state *j* on reading the symbol *a* in the alphabet. The final state probability *P*_*if*_ is the probability that the string will end at state *q*_*i*_, such that the following constraints applies:

$$p_{if}+\sum_{q_{j}\in Q}\sum_{a\in \Sigma }p_{ij}(a)=1$$ (1)

Eq 1 signifies the fact that the total probability of each transition out of the state *q*_*i*_ together with the probability that *q*_*i*_ is an accepting state, must be 1.

The probability that the string *w* to be generated by A is defined as

$$p(w)=\sum_{q_{j}\in Q}p_{1j}(w)p_{if}$$

$$p_{ij}(w)=\underset{q_{k}\in Q}{\sum }\underset{w\in \Sigma }{\sum }p_{ik}(b)p_{kj}(a)\text{ where }ba\text{ = }w$$ (2)

The language generated by the automaton A is the stochastic regular language define as:

$$L=\{w\in \Sigma^{*}:p(w)\neq 0\}$$ (3)

This means that the set of sequences that have a nonzero probability will be accepted by the DSFA.

In the first step of constructing the DSFA corresponding to the phosphorylation sites, the training and test data set is created which are mutually exclusive. Training set consists of only positive samples, i.e., the sequences having phosphorylation sites of each kinase. Test set contains both the positive and negative samples. In the next step, a PTA is created using the training dataset. A PTA is a tree-like DFA generated by extracting all the prefixes of the sample sequences as states that accepts only the sample sequences from which the PTA is built. Given a set of random sequence of symbols (e.g., *w = a*_*1*_ *a*_*2*_ *a*_*3*_ *a*_*4*_...*a*_*n*_), a prefix of the sequence *w* can be any subsequence *w*_*p*_ of *w* for which:

$$w=w_{p}w’$$

where *w*’ is a sequence of any length (i.e., containing any number of symbols, including 0). A prefix tree, also known as *trie*, (the term is derived from the word retrieval, representing an ordered multi-way tree data structure conferring much advantage over a binary tree to store strings over an alphabet) is an ordered tree data structure and it is expressed as a DFA to form the PTA. If S be the sample from which we have built the PTA, a PTA = PTA(S) is a DFA that contains a path from the initial state to a final accepting state for each strings in S.

PTA can be reduced in size by merging the equivalent states of PTA to form a final DSFA. In the next step of our method, we have generated the deterministic stochastic finite state automata (DSFA) from the PTA using a well known GI algorithm, Alergia proposed by R.C. Carrasco and J Oncina [44]. Alergia transforms a PTA into a DSFA by means of a systematic merging of statistically equivalent states. Alergia takes PTA as an input and evaluates the relative frequencies of the outgoing edge of each state of the PTA and are calculated by estimating the following parameters:

- *n*_*i*_, the number of strings arriving at state *q*_*i*_,
- *f*_*i*_ *(a)*, the number of strings following edge *δ*_*i*_ *(a)*
- *f*_*i*_ *(#)*, the number of strings ending at node *q*_*i*_,

$\frac{f_{i}(a)}{n_{i}}$ and *f*_*i*_(#) estimates the *p*_*i*_ *(a)* from the state *q*_*i*_ while reading the symbol *a* and the ending probability respectively *p*_*if*_.

In the next step, the Alergia algorithm compares the pairs of states (*q*_*i*_,*q*_*j*_) with 1 ≤ *i* ≤ *j* -1 and 2 ≤ *j* ≤ *t*, where t is the total number of states in PTA. Two states are said to be equivalent if they have equal outgoing transition probability for each symbol *a* ϵ Σ and the same destination states. Therefore, the criterion for two states to be equivalent is given by the following equation:

$$q_{i}\equiv q_{j}\Rightarrow \{\begin{array}{c} p_{i}(a)\equiv p_{j}(a)\\ \delta_{a}(i)\equiv \delta_{a}(j) \end{array},\forall a\in \Sigma$$ (4)

However, the determination of state equivalence is statistical in nature and is controlled by a parameter α, called the confidence parameter that ranges between 0 and 1. Confidence parameter is introduced to overcome any possible statistical fluctuations in the experimental data. Two states are merged when they are equivalent within some tolerance limits defined in terms of confidence range. The state transition probabilities and final state probabilities are recomputed after each state merging. The algorithm is guaranteed to converge to the target DSFA in the limit when a complete sample is provided. The detail of the Alergia algorithm is given in the original paper by Carrasco *et al*. [44]. In our method, we have assumed that there exists at least some non-zero probability of transition from each state for each symbol in Σ. Therefore if the transition probability and ending probability for some states is 0, we have replaced the corresponding probability by a small non-zero value, i.e., by 0.01.

Once the DSFA is constructed using the training set, the probability of a specific sequence is generated by the DSFA using Eq 2. If the probability of a given sequence is above the user defined threshold value, the sequences are predicted to be phosphorylation sites.

### Extraction of probabilities of specific pattern

Once the DSFA is constructed, useful information, such as probability of appearance of a particular symbol in a random sequence can be extracted from it. As the input protein sequence does not provide any information regarding appearance of phosphorylated residues, it would be troublesome to decide which one of the S/T residues will be the exact phosphorylated residue if S/T appears more than once in a sequence. To overcome this problem, we have calculated the probability of occurrence of S/T residues to infer the exact position information of the phosphorylated residue in a random sequence. The probability of a particular symbol to appear in any given sequence can be calculated from the transition probability matrix *P*_*ij*_ *(a)*. A path starting from S and containing any symbol *x* either begins with the symbol *x* or begins with some other symbol and is followed by a path starting at the next state containing a symbol *x*. Therefore, the probability of occurrence of the symbol *x* can be written as *P*(*S*,*x*) where S can be a start state or any state following the start state which emits the symbol *x*.

Thus, for each state, the emission probability of each S/T symbol is calculated and the S/T residue for which emission probability from a state is highest is considered to be a putative phosphorylated residue and the position of that S/T residue in the sequence is predicted as phosphorylation site specific to a given kinase.

### Performance evaluation

The performance of our method is measured in terms of four generalized statistical parameters i.e., precision, recall, accuracy (AC) and F-measure. The measures are given by the following equations:

Recall=TPTP+FN, Precision=TPTP+FP, AC=TP+TNTP+FP+TN+FN

F−measure=2*Precision*RecallPrecision+Recall

where TP is the true positive i.e., positive instances predicted as positives, TN is the true negative i.e., negative instances predicted as negative, FP or false positive is the negative instances predicted as positives and FN is false negative i.e., positive instances predicted as negative.

Receiver operating characteristic (ROC) curves are calculated and plotted based on Sensitivity(Sn) and Specificity (Sp)to evaluate the prediction performance of our method for various threshold. S_n_ and S_p_ are calculated by the following equations:

$$S_{n}=\frac{TP}{TP+FN}\;and\;S_{p}=\frac{TN}{TN+FP}$$

S_n_ and S_p_ values depend on the threshold used for the prediction. A higher threshold improves S_p_ but reduce S_n_, whereas lesser threshold increases the S_n_ at the price of lower S_p_.

## Results and Discussion

### Performance evaluation of our method on various datasets

We consider the prediction of phosphorylation site to be an automata problem. For such a problem, given an amino acid sequence as input to the automata, our system accepts the sequences having phosphorylation sites and rejects the sequences lacking the phosphorylation sites. This is done by generating a probability corresponding to the input sequence. If the probability is above the user defined threshold, the sequence is predicted to be a phosphorylation site.

**Fig 4:**
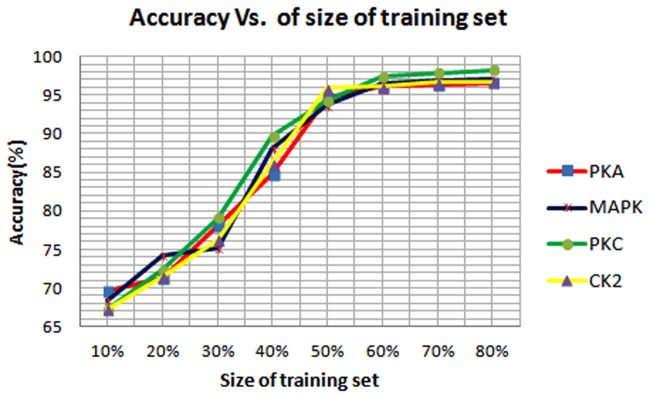
Change of accuracy with various size of training set (10%-80%) for the Alergia algorithm.

**Fig 5:**
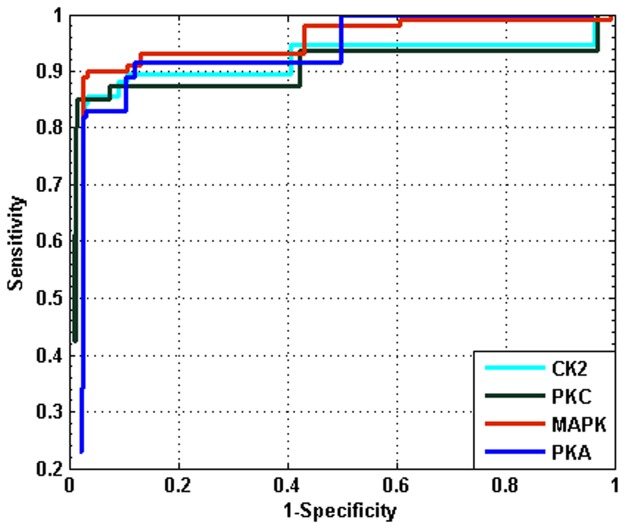
ROC curve by varying threshold values for PHSDB dataset for the four kinases PKA, PKC, MAPK and CK2.

Positive and negative sequences for each kinase family are downloaded from PHOSPHO.ELM database and CDHIT program is run on these sequences to obtain the non-homologous dataset. Next, the positive data is divided into training and test datasets. We varied the size of the training set by taking 10% through 80% of the whole positive data. The accuracy of our method with various training data sizes is shown in Fig 4. We found from the Fig 4, that a 60% training size is sufficient to achieve the best accuracy. Hence, we have divided the dataset into 6:4 ratios, where 60% data are used as training dataset for constructing the DSFA for further explanation and comparison purpose. Taking the remaining 40% data and the negative sequences, a test dataset is constructed. In test dataset, the ratio of positive and negative sequences is kept at 1:10 to avoid any biased prediction. We have named this dataset PHSDB. The number of positive and negative samples in training and test dataset for four kinases are summarized in Table 1. Furthermore, we have varied threshold value from 0.001 to 0.01 for all the kinases. We have varied the window size from 5 to 21 and have found that for window size 7 our proposed method obtains best result. Hence we have fixed the window size of our method at 7. In order to evaluate the performance of our method, we have generated Receiver Operating Curve (ROC) for each of the kinase specific phosphorylation site predictor. ROC curve shows the trade-off between True Positive Rate (TPR) i.e., Sensitivity and False Positive Rate (FPR) i.e., 1-Specificity. ROC curve is obtained by varying the threshold from 0.01 to 0.004. The ROC curve for PHSDB dataset is shown in Fig 5. The ROC curves for all the kinases almost reach100% sensitivity with atleast 15% specificity. Moreover, from the Fig 5 we can see that for all the kinases, at the threshold value nearer to 0.008, a balanced and higher sensitivity and specificity is obtained. While decreasing the threshold, the corresponding specificity also decreases. Hence for the further experimentation we have used the threshold value 0.008 for our method. We have also calculated the precision, recall, accuracy and F-measure for PHSDB dataset for the threshold 0.008 as shown in Table 2. The table shows that our method can predict phosphorylation sites for PKA, PKC, MAPK and CK2 with 96.11%, 97.38%, 96.54% and 96.12% accuracy respectively and also quite high F-measure values of 0.7933, 0.8553, 0.8240 and 0.7999 respectively. It indicates that our method yields sufficiently high precision and recall values which in other term means that our proposed approach can predict positive and negative instances quite accurately. To validate our result from ROC curve that at threshold value 0.008 yields the best result, we have also plotted change in accuracy with various threshold values. The plot is shown in S1–S4 Figs. demonstrates that at threshold value 0.008 our method obtains best accuracy.

**Table 1:** Number of positive and negative samples in training and test dataset for four protein kinases after removing the redundant data obtained from Phospho.ELM database version 9.0.

Name of the kinase	Training dataset	Test dataset	
	Positive samples	Positive samples	Negative samples
**PKA**	168	117	1172
**PKC**	115	80	801
**MAPK**	144	102	1013
**CK2**	109	76	751

**Table 2:** Performance of the proposed method for prediction of phosphorylation site specific to the kinases PKA, PKC, MAPK, CK2 on the training and test dataset.

Name of the kinases	Precision	Recall	F-measure	Accuracy(%)
	PHSDB	PHSDB	PHSDB	PHSDB
**PKA**	0.7680	0.8205	0.7933	96.11
**PKC**	0.8607	0.8500	0.8553	97.38
**MAPK**	0.7672	0.8900	0.8240	96.54
**CK2**	0.7529	0.8533	0.7999	96.12

Here 60% of the positive data is used for training and the remaining 40% Data along with negative data is used as test dataset. We renamed this test dataset as PHSDB.

As we have very limited number of known phosphorylation sites corresponding to the specific kinases, we have adopted an asymmetric bootstrap resampling approach for the dataset containing only the positive phosphorylation sites. This procedure generates more than one numbers of positive datasets for better statistical validation. In bootstrap resampling, a set of resampled subsets of the original dataset are generated by random sampling with replacement (so that individual instances may appear more than once in a subset). The size of the resampled subsets may or may not be equal to the size of the original dataset. In our case, we have kept the size of the subsets equal to the original one. That is, from the original dataset D of size n, a set of new datasets {D_1_, D_2_, D_3_, …, D_m_} are generated each of size n’ such that n = n’. We have varied the number of resampled subsets which (i.e., B) from 10 to 50. The results of repeating each experiment for 30 times with each of the resampled dataset for each kinase is reported in Table 3. In order to obtain a statistically significant output for testing our proposed method, we have done a 10-fold cross-validation on each of the resampled dataset. In 10-fold cross-validation, the dataset is divided into 10 equal sized parts. 9 parts are used for training and the remaining part is used as test dataset. The method is repeated for all the 10 parts. In each test dataset, the ratio of positive samples to negative samples is kept at 1:10. Table 3 shows an improvement in the result for all the kinases in terms of both accuracy and F-measure. Here also, our method performs quite satisfactory with accuracy in the range of 96%-98% and F-measure in the range of 0.83–0.88 for all the resampled datasets and for all the kinases. Table 3 shows that a very small variation in precision, recall, accuracy and F-measure is found in the kinases PKA, PKC, MAPK and CK2 with the change of B. It might be possible that the presence of some atypical samples in these families increases the difference between various re-sampled datasets and causing the small variation in accuracy and F-measure with the different numbers of re-sampled datasets; but none of these kinase families show any trend of increase or decrease of accuracy with increase or decrease of re-sampled data size Hence, the performance of the method does not get affected by the number of the re-sampled datasets. Table 3 shows a balanced Precision and recall indicating that the number of true positives and true negatives is higher relative to the number of false positives and false negatives. As a result, quite a high F-measure has resulted for all the kinases using our method. ROC curves obtained by varying threshold values for various resampled datasets corresponding to all the kinases are shown in S5–S9 Figs.

**Table 3:** Performance of our method on different resampled data sets for the kinases PKA, PKC, MAPK, CK2.

Name of the Kinase	Number of resampled dataset	Precision	Recall	Accuracy(%)	F-measure
**PKA**	B = 10	0.8012	0.8958	96.73	0.8459
	B = 20	0.7981	0.8784	96.56	0.8363
	B = 30	0.7969	0.8993	96.70	0.8450
	B = 40	0.8012	0.9097	96.83	0.8520
	B = 50	0.8018	0.8993	96.76	0.8477
**PKC**	B = 10	0.8483	0.8950	98.04	0.8710
	B = 20	0.8495	0.8750	97.93	0.8620
	B = 30	0.8599	0.8900	98.11	0.8746
	B = 40	0.8529	0.8700	97.93	0.8613
	B = 50	0.8599	0.8900	98.11	0.8746
**MAPK**	B = 10	0.7889	0.9230	96.77	0.8507
	B = 20	0.7800	0.9190	96.61	0.8438
	B = 30	0.7876	0.9311	96.81	0.8534
	B = 40	0.7857	0.9352	96.81	0.8539
	B = 50	0.7842	0.9271	96.73	0.8497
**CK2**	B = 10	0.8076	0.8983	96.86	0.8506
	B = 20	0.8067	0.8930	96.80	0.8477
	B = 30	0.8019	0.8877	96.70	0.8426
	B = 40	0.8078	0.8770	96.70	0.8410
	B = 50	0.8086	0.9037	96.91	0.8535

Here we have varied the size of resampled data sets from 10 to 50. The performance is measured in terms of precision, recall, accuracy and F-measure.

### Performance Comparison with other GI methods

We have also considered two other popular grammar inference algorithms: RPNI based on ordered depth-first search named regular positive and negative inference (RPNI) [45, 46] and another is based on genetic algorithm [47].

RPNI performs an ordered depth first search to identify a DFA in polynomial time using dataset S consisting of both the positive and negative examples *S* = (*S* ^+^ ∪ *S* ^-^). RPNI algorithm first constructs a PTA for *S* ^+^. The states of the PTA are then numbered in standard order. The set of string that would lead from start state to each individual state of the PTA is determined. The strings are sorted in lexicographical order. Each state is numbered based on the position of the corresponding string in the sorted list. Then the states of the PTA are systematically merged using a quadratic loop i.e., in each step i, the state q_i_ is merged with the states *q*_0_,*q*_1_,*q*_2_,…,*q*_*i*-1_ in order. If the quotient automaton obtained by merging any two states does not accept any instance belonging to *S* ^-^, the quotient automaton is treated as the current target and the search for a more general solution is continued with the state *q*_*i*-1_.

Genetic algorithm provides an attractive framework and has been popularly used to infer the DFA. A typical genetic algorithm based search involves evolving of randomly generated set of individuals based on the survival of the fittest principle of Darwinian evolution. Here a PTA is constructed from the positive sample set *S* ^+^. In genetic algorithm, the initial population comprises of a random selection of elements from the set of partitions of the set of states of the PTA. Each element of the initial population is a quotient automaton belonging to the lattice of FSA constructed from PTA. The fitness of each quotient FSA is represented as the function of two variables: the number of states of the FSA and the number of instances in *S* ^-^, which are misclassified by the FSA. Highest fitness is assigned to the individuals having fewer states and making fewer errors. After each generation, a subpopulation of individuals is randomly selected for reproduction based upon their fitness values. Two genetic operators- mutation and crossover- are applied to a subpopulation to produce an offspring. The numbers of offspring produced by the genetic reproduction are valid partitions belonging to the lattice. These are added to the original population. A fitness-proportionate selection scheme is used to randomly select individuals for the next generation. After a pre-specified number of generations, the fittest individual from the population is selected and the algorithm returns the inferred FSA. Although this approach does not necessarily guarantee for convergence to the target DFA, experimental results have shown that this method is able to identify adequately small DFAs that make reasonably small errors in differentiating positive and negative sequences.

The above two methods give rise to simple deterministic finite state automata (DFA) whereas Alergia algorithm infers a deterministic stochastic finite state automata (DSFA). To compare the performance of the three GI algorithm, we performed 10 independent choices of training and test set where the training set consists of 60% of the positive sequences and the test set the remaining 40%. The three GI algorithms are then run on these generated datasets, so that for each run the selection of training and testing sets are same for all the methods.

**Fig 6:**
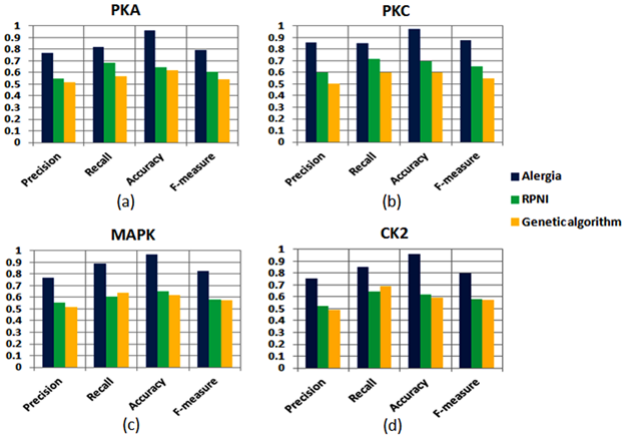
Performance of the Alergia algorithm in comparison to the other two grammar inference methods (RPNI and Genetic algorithm) in terms of precision, recall, accuracy and F-measure for kinases: (a)PKA, (b)PKC, (c)MAPK and (d)CK2.

The comparison results of these three grammar inference methods in terms of precision, recall, accuracy and F-measure for all the kinases are shown in Fig 6A–6D According to the Fig 6, we obtain that the Alergia algorithm performs best in terms of all these parameters for all the kinases among the three GI algorithms. RPNI and genetic algorithm performs almost equally well although genetic algorithm performs slightly better than RPNI for predicting the true positives among total positives, i.e., yield a better recall value for MAPK and CK2 kinase. For all the other parameters and kinases, RPNI performs superior to genetic algorithm.

To test whether the prediction accuracy of the our proposed method based on Alergia algorithm, is significantly higher than those of other GI method based on RPNI and Genetic algorithm, a paired t-test was performed on the accuracy results obtained from the datasets. The null hypothesis is that the difference between the means of accuracies obtained by the two methods is zero and the alternative hypothesis is that the difference is positive. Table 4 summarizes the average accuracy returned by each GI method on the 10 datasets for all the four kinases. Table 4 clearly shows that Alergia algorithm yields highest accuracy as compared to other GI methods for all the kinases. A hypothesis test is performed to evaluate the significance of the differences between the performances of the Alergia algorithm with other two GI methods. The results at 5% significance level are summarized in Table 5. From the Table 5 we find that the null hypothesis is rejected at 5% significance level for the problem in hand. It means that the prediction accuracy rates obtained using our proposed method using Alergia algorithm are higher than those obtained using other two GI methods and the difference is statistically significant. It is also noted that, all P-values obtained through paired t-test are smaller than 0.01, which means that the differences are statistically highly significant.

**Table 4:** Average accuracy of three different GI algorithms (Alergia, RPNI, Genetic Algorithm) on 10 various test sets generated by us.

Name of the Kinase	Name of the GI method name	Accuracy (%)
**PKA**	Alergia	95.68
	RPNI	65.07
	Genetic Algorithm	61.79
**PKC**	Alergia	97.91
	RPNI	70.1
	Genetic Algorithm	60.49
**MAPK**	Alergia	96.84
	RPNI	65.35
	Genetic Algorithm	61.94
**CK2**	Alergia	96.18
	RPNI	62.29
	Genetic Algorithm	59.28

**Table 5:** The results of t-test at 5% significance level for various GI methods for PKA, PKC, MAPK and CK2.

Name of Kinase	GI Method	T-value	P-value	Null Hypothesis
**PKA**	Alegia Vs RPNI	157.27	1.05E-29	Reject
	Alegia Vs GA	195.93	2.02E-31	Reject
**PKC**	Alegia Vs RPNI	199.03	1.53E-31	Reject
	Alegia Vs GA	294.51	1.32E-34	Reject
**MAPK**	Alegia Vs RPNI	262.69	1.03E-33	Reject
	Alegia Vs GA	291.10	1.63E-34	Reject
**CK2**	Alegia Vs RPNI	262.17	1.07E-33	Reject
	Alegia Vs GA	278.88	3.53E-34	Reject

### Performance Comparison with other phosphorylation site prediction methods

In order to evaluate the performance of our proposed method, we have compared our approach with five other popular open access kinase specific phosphorylation site prediction methods along with our previous proposed method. In most of the previous studies, these five methods have been used because of their high performance and free availability in the public domain. The six methods are PPSP [10], KinasePhos2.0 [8], GPS2.1 [11, 48], Scansite [7], NetphosK 1.0 [9] and our previous method [17]. PPSP is based on Bayesian decision theory (BDT). Kinasephos uses Hidden Markov Model (HMM) to predict kinase specific phosphorylation sites. Scansite searches for motifs within proteins that are likely to be phosphorylated by specific protein kinases, using the scores calculated from position-specific score matrices (PSSM). Netphosk1.0 employs an artificial neural network to predict 17 kinase-specific phosphorylation sites while GPS2.1 server uses a modified version of group based scoring algorithm [49, 50] to predict PK specific phosphorylation sites in hierarchy. Our previous method employs an ensemble method approach to predict kinase specific phosphorylation sites. Here, we have opted predict by individual kinase and balanced performance option for PPSP. In the case of NetphosK, prediction without filtering and a threshold value of 0.5 was selected to predict phosphorylation sites. KinasePhos2.0 was run with the option of default specificity for a specific kinase. In this work, Scansite2.0 was run by searching all motifs and the “medium stringency level” setting was selected. For GPS2.1, a medium threshold was selected for a particular kinase family.

**Fig 7:**
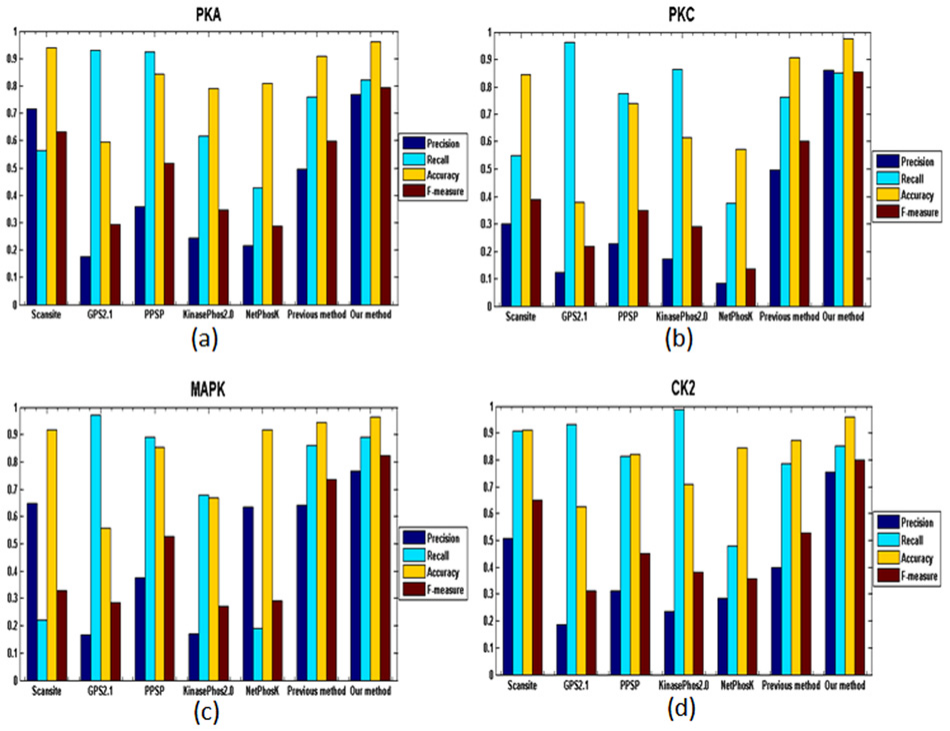
Performance comparison of six methods along with our proposed method in terms of precision, recall, accuracy, F-measure for the four types of kinases: (a)PKA, (b)PKC, (c)MAPK and (d)CK2.

To avoid biased prediction, we have considered a candidate sequence to be true positive only when the sequence is predicted correctly. We have used the sequences in the 40% test dataset, i.e. PHSDB dataset taken from the PhosPho.ELM database version 9.0 for comparison. The advantage of using the PHSDB dataset is its non-biasness and independence; thereby we can fairly compare several existing methods with our proposed method. The performance of comparison is assessed on the basis of the parameters precision, recall, accuracy and F-measure. Fig 7A–7D shows the comparisons of predictive performance of our method with the six other prediction systems for the Kinase PKA, PKC, MAPK and CK2 respectively. From the Fig 7 we observed that our method outperforms all the methods in terms of Precision, Accuracy and F-measure for all the kinases. For kinase CK2, KinasePhos2.0 yields the highest recall value followed by GPS2.1 and for all the other kinases (PKA, PKC and MAPK) GPS2.1 yields superior recall values. NetPhosK performs worst in terms of recall for all the kinases. PPSP performs better in terms of recall but yield a lower precision value. GPS2.1 performs worst in terms of precision for PKA, MAPK and CK2. NetPhosK obtains lowest precision for PKC. Fig 7A–7D shows that for Kinase PKA, PKC, MAPK and CK2, the six methods achieve a good recall value but sacrificing the precision result in a low F-measure. Also lower precision value implies a higher number of false positives. GPS 2.1 and PPSP obtain a very high recall value but at the same time a very low precision value which means these two methods have yielded a large number of false positives. For PKA and CK2, GPS2.1 performs worst in terms of accuracy and F-measure whereas NetPhosK lowest accuracy and F-measure for PKC and KinasePhos 2.0 for MAPK. Our method offers high and balanced precision as well as recall, which reflects that our method is superior to the well known existing phosphorylation site prediction methods and can effectively distinguish the phosphorylation sites from non-phosphorylation sites in a kinase specific manner. Moreover, the good performances of our method illustrates that our method can efficiently evaluate the sequence similarity of phosphorylation substrates for different kinases.

## Conclusion

In this article, we have proposed a novel kinase specific phosphorylation prediction method based on primary sequence information only. The method is simple because it does not require any sequence encoding and hence computational complexity is reduced. Experimental results of the method show a satisfactory performance as compared to other methods and quite a good performance improvement over our previous method for all the kinases. Alergia algorithm infers Deterministic stochastic finite state automata that perform better than the finite state automata inferred by RPNI and genetic algorithm. In summary, applying the new method produces good results without the need for a sophisticated machine learning techniques in detecting phosphorylation sites. Furthermore, the results of the predictions through the proposed GI based method indicate that our method is very promising in detecting protein phosphorylation sites and may play an important complementary role against existing methods. We expect to apply our new method to other kinds of biological systems to achieve high performance and a substantial improvement.

## Supporting Information

S1 Fig Change of accuracy with various threshold values for the kinase PKA. (TIF)

S2 Fig Change of accuracy with various threshold values for the kinase PKC. (TIF)

S3 Fig Change of accuracy with various threshold values for the kinase MAPK. (TIF)

S4 Fig Change of accuracy with various threshold values for the kinase CK2. (TIF)

S5 Fig ROC curve by varying threshold for four kinase PKA, PKC, MAPK and CK2 for resampled dataset size B = 10. (TIF)

S6 Fig ROC curve by varying threshold for four kinase PKA, PKC, MAPK and CK2 for resampled dataset size B = 20. (TIF)

S7 Fig ROC curve by varying threshold for four kinase PKA, PKC, MAPK and CK2 for resampled dataset size B = 30. (TIF)

S8 Fig ROC curve by varying threshold for four kinase PKA, PKC, MAPK and CK2 for resampled dataset size B = 40. (TIF)

S9 Fig ROC curve by varying threshold for four kinase PKA, PKC, MAPK and CK2 for resampled dataset size B = 50. (TIF)

S1 Dataset List of training sequences for PKA. (TXT)

S2 Dataset List of training sequences for PKC. (TXT)

S3 Dataset List of training sequences for MAPK. (TXT)

S4 Dataset List of training sequences for CK2. (TXT)

S5 Dataset List of test sequences PKA. (TXT)

S6 Dataset List of test sequences PKC. (TXT)

S7 Dataset List of test sequences MAPK. (TXT)

S8 Dataset List of test sequences CK2. (TXT)
