# Supplementary figures and images for: A Grammar Inference Approach for Predicting Kinase Specific Phosphorylation Sites
Source: PLoS One. 2015 Apr 17;10(4):e0122294. doi: 10.1371/journal.pone.0122294 (PMC4401752; doi:10.1371/journal.pone.0122294)

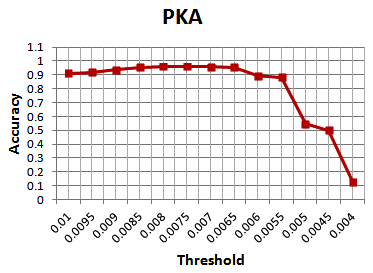

Supplement: S1 Fig — (TIF) [file pone.0122294.s001.tif]

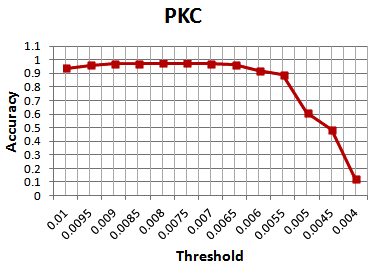

Supplement: S2 Fig — (TIF) [file pone.0122294.s002.tif]

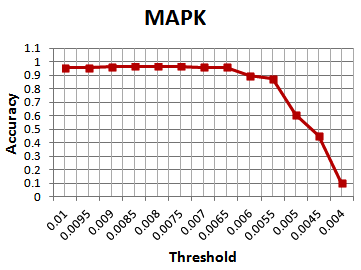

Supplement: S3 Fig — (TIF) [file pone.0122294.s003.tif]

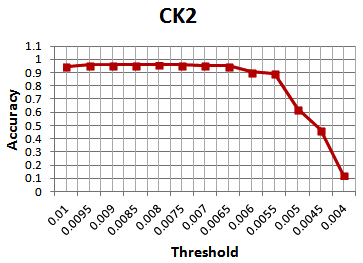

Supplement: S4 Fig — (TIF) [file pone.0122294.s004.tif]

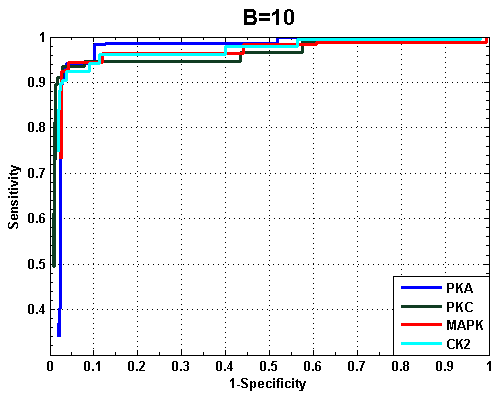

Supplement: S5 Fig — (TIF) [file pone.0122294.s005.tif]

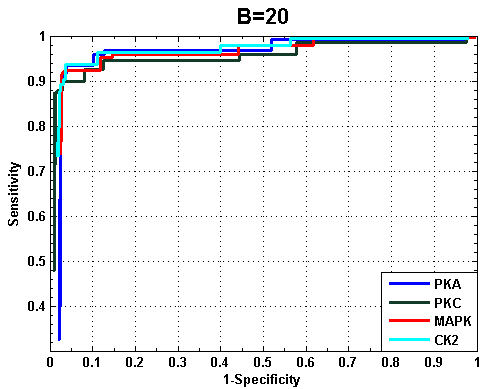

Supplement: S6 Fig — (TIF) [file pone.0122294.s006.tif]

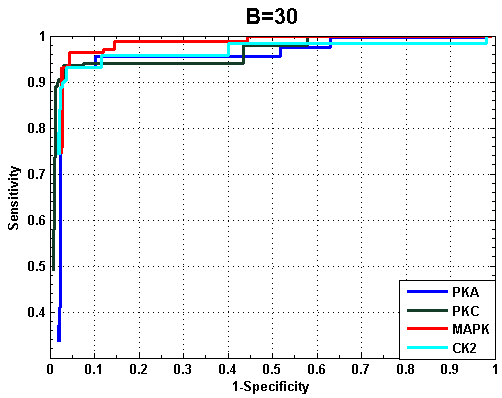

Supplement: S7 Fig — (TIF) [file pone.0122294.s007.tif]

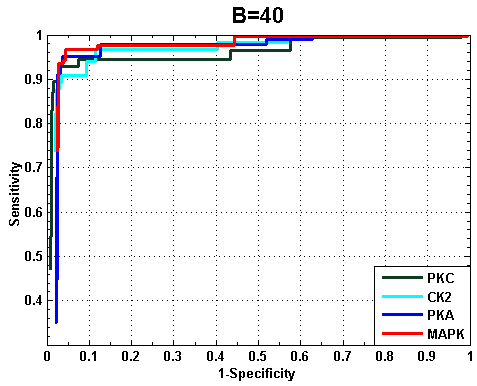

Supplement: S8 Fig — (TIF) [file pone.0122294.s008.tif]

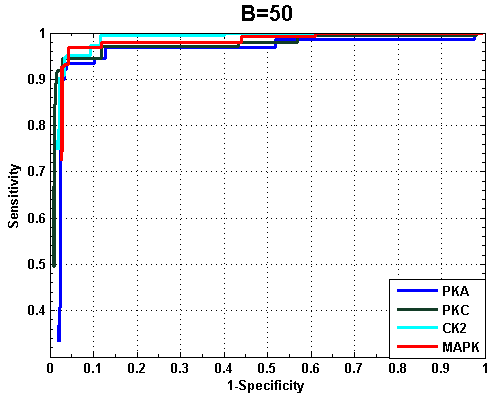

Supplement: S9 Fig — (TIF) [file pone.0122294.s009.tif]
